# Microscopic observation of the effects of elongation on the polymer chain dynamics of crosslinked polybutadiene using quasi-elastic γ-ray scattering

**DOI:** 10.1107/S1600577522007998

**Published:** 2022-08-23

**Authors:** Ryo Mashita, Makina Saito, Yoshitaka Yoda, Hiroyuki Kishimoto, Makoto Seto, Toshiji Kanaya

**Affiliations:** a Chemical Analysis Center, Sumitomo Rubber Industries Ltd, Tsutsui-cho, Chuo-ku, Kobe, Hyogo 651-0071, Japan; b Department of Physics, Graduate School of Science, Tohoku University, Sendai, Miyagi 980-8578, Japan; c Japan Synchrotron Radiation Research Institute, Sayo, Hyogo 679-5198, Japan; d Institute for Integrated Radiation and Nuclear Science, Kyoto University, Sennan, Osaka 590-0494, Japan; e Kyoto University, Japan; NSRRC, Taiwan

**Keywords:** polymer dynamics, quasi-elastic γ-ray scattering, elongation

## Abstract

A synchrotron-radiation-based quasi-elastic γ-ray scattering system has been developed that uses time-domain interferometry to observe microscopic polymer dynamics under uniaxial deformation. The stress-producing mechanism of crosslinked polybutadiene has been studied from a microscopic viewpoint. It was found that the mean relaxation time 〈τ〉 of the microscopic polymer motion observed over a relatively high temperature (*T*) range (*i.e.* *T* ^−1^ < 0.0045 K^−1^) increased with elongation on both the intra- and intermolecular scales. Following an extensive strain dependence study, it was found that the strain dependences of both the intra- and intermolecular 〈τ〉 changed with the stress dependence. It was therefore suggested that 〈τ〉 increased due to the constraint of the local polymer chain motion caused by elongation. The local molecular dynamics of polymer chains under uniaxial deformation could be evaluated at intra- and intermolecular scales separately for the first time using our method.

## Abstract 2

A synchrotron-radiation-based quasi-elastic γ-ray scattering system has been developed that uses time-domain interferometry to observe microscopic polymer dynamics under uniaxial deformation. The stress-producing mechanism of crosslinked polybutadiene has been studied from a microscopic viewpoint.

## 1. Introduction

Rubber materials are indispensable in daily life due to their high mechanical strength, resistance to fracture and resistance to light deterioration, which allows their wide application in various products. Improvements in the mechanical strength and resistance to fracture are required in numerous rubber products. It is therefore desirable to clearly understand the microscopic fracture mechanism of rubber, and so considerable research has been conducted on the fracturing of polymer materials (Ishikawa & Chiba, 1990 ▸; Bayraktar *et al.*, 2008 ▸; Uesugi *et al.*, 2016 ▸; Mashita *et al.*, 2021 ▸; Choi *et al.*, 2012 ▸; Naoki *et al.*, 1974 ▸; Nakajima *et al.*, 1978 ▸; Diaz-Calleja *et al.*, 1987 ▸; Munch *et al.*, 2006 ▸; Boyer, 1968 ▸; Engels *et al.*, 2012 ▸; Retting, 1970 ▸; Kalfoglou & Williams, 1972 ▸; Suzuki *et al.*, 2019 ▸). For example, it was reported that the cavities formed during the fracture of a material improve its resistance to further fracture owing to the release of polymer constraints between cavities (Ishikawa & Chiba, 1990 ▸). Although the macroscopic fracture phenomenon has been examined using electron microscopy, X-ray tomography and mechanical measurements (Bayraktar *et al.*, 2008 ▸; Uesugi *et al.*, 2016 ▸; Mashita *et al.*, 2021 ▸), few studies have focused on the changes in polymer dynamics associated with stress concentration and release at the molecular scale. Understanding the stress-producing phenomenon over a wide range (*i.e.* from the macroscopic to the microscopic scale) is therefore necessary to elucidate the mechanism of stress concentration and release.

Previously, the dynamics of diffusional α-relaxation at the molecular scale under an external force were evaluated using dielectric relaxation spectroscopy measurements to examine the dynamics of polymers under uniaxial deformation (Choi *et al.*, 2012 ▸; Naoki *et al.*, 1974 ▸; Nakajima *et al.*, 1978 ▸; Diaz-Calleja *et al.*, 1987 ▸; Munch *et al.*, 2006 ▸). To date, a variety of tensile effects have been reported in the context of molecular dynamics, including the fact that a decrease in the configurational entropy due to chain orientation leads to longer relaxation times (Naoki *et al.*, 1974 ▸; Nakajima *et al.*, 1978 ▸), the observation that a small decrease in the polymer density tends to speed up the dynamics (Diaz-Calleja *et al.*, 1987 ▸), and the fact that elongation has no effect on the polymer dynamics (Munch *et al.*, 2006 ▸). Despite these results, a unified theory of the relationship between stress production and molecular dynamics has yet to be developed owing to the lack of spatial information obtained by previous dielectric relaxation spectroscopy measurements. Thus, to understand the effects of stress on molecular dynamics in more detail, it is necessary to elucidate the relationship between the dynamics and the structure on the molecular scale by determining the spatial scale. The Johari–Goldstein (JG) β-relaxation process, which is a local activation process on the molecular chain scale, is related to the mechanical properties of various types of polymers (in deeply supercooled and glass states) and has been examined based on the results of dynamic viscoelastic measurements (Boyer, 1968 ▸; Engels *et al.*, 2012 ▸; Retting, 1970 ▸; Kalfoglou & Williams, 1972 ▸; Suzuki *et al.*, 2019 ▸). However, the mechanism of local confinement of the polymer chain, which is regarded as the origin of stress production, remains unclear.

Quasi-elastic scattering methods, which can determine the time–spatial scale of microscopic polymer relaxation on the molecular scale, are useful for the study of polymer materials (Arrighi *et al.*, 1998 ▸; Chrissopoulou *et al.*, 2007 ▸; Roh *et al.*, 2013 ▸). In our previous report, polymer dynamics were measured on the nanosecond timescale, wherein the scattering vector *q* was set in the range 1.2–18 nm^−1^ using quasi-elastic neutron scattering (*q* = 4πsinθ/*l*, where 2θ and *l* are the scattering angle and the wavelength of the neutron, respectively) to reveal the relationship between the elastic modulus of the polymer material and the heterogeneous polymer dynamics (Mashita *et al.*, 2017 ▸). Furthermore, through the use of quasi-elastic γ-ray scattering (QEGS) with time-domain interferometry (TDI), which can detect energy resolutions on the order of nano eV, the dynamics of various polymer materials have been measured in the time range of nano- to microseconds over a *q* range of 10–60 nm^−1^ (Saito *et al.*, 2012 ▸; Kanaya *et al.*, 2014 ▸). Using QEGS to examine non-crosslinked polybutadiene, α-relaxation and JG β-relaxation were observed in the spatial range corresponding to the first and second peaks of the structural factor, respectively. This result indicates that different relaxation processes can be detected separately when they are separated into different spatial ranges (Saito *et al.*, 2012 ▸; Kanaya *et al.*, 2014 ▸). From an industrial viewpoint, the effect of the addition of silica nanoparticles to non-crosslinked polybutadiene has also been investigated, and it was found that the α-relaxation and JG β-relaxation motions of the polymer became slower in the presence of these nanoparticles (Saito *et al.*, 2017*a* ▸, 2021 ▸). These previous studies therefore indicate that QEGS is a powerful method for investigating polymer dynamics on the molecular scale. However, the above studies were conducted under non-deformation conditions, and, in order to understand the stress-producing phenomenon on the molecular scale, dynamic measurements under deformation are necessary.

Thus, we herein report the development of a method for the measurement of polymer dynamics under uniaxial deformation using QEGS, followed by a systematic investigation of the relationship between the microscopic polymer dynamics and the stress–strain behavior at each characteristic molecular spatial scale. For this purpose, the temperature dependences of the dynamics of crosslinked and non-crosslinked polybutadiene were initially measured to elucidate the effect of crosslinking on the polymer dynamics. Subsequently, the temperature dependences of the α- and JG β-relaxation times of the crosslinked polybutadiene were measured under uniaxial deformation to elucidate the effect of deformation on the polymer dynamics using the developed QEGS method. Finally, the strain dependence of the polymer dynamics was measured systematically to understand the relationship between polymer dynamics and stress–strain behavior. Such measurements under external conditions, such as stress, magnetic field and thermal, are essential for studies with material development as output and for the currently increasing demands of the industry. With this achievement as a springboard, further developments in polymer dynamics measurements under various kinds of external conditions are expected.

## 2. Experimental

### 2.1. Materials and sample preparation

Polybutadiene (PB; NF35R, Asahi Kasei Co. Ltd) with a weight-average molecular weight (*M*_w_) of 3.5 × 10^5^ and a molecular weight distribution index (*M*_w_/*M*_n_) of 2.3 was employed for the purpose of this study. It should be noted that *M*_n_ is the number-average molecular weight of the polymer matrix. The percentages of *cis*, *trans* and vinyl PB isomers were 35, 52 and 13%, respectively. Sulfur powder (Tsurumi Chemical Industry Co. Ltd) was used as the crosslinker and *N*-cyclo­hexyl-2-benzo­thia­zole sulfonamide (NOCCELER CZ, Ouchi Shinko Chemical Industrial Co. Ltd) was used as the accelerator for the crosslinking reaction.

For sample preparation, PB, the sulfur powder and *N*-cyclo­hexyl-2-benzo­thia­zole sulfonamide were mixed in a weight ratio of 100:0.95:0.95 using a six-inch two-roll mill for 5 min at 298 K. Subsequently, the mixture was placed in a mold and heated at 443 K for 40 min to increase the rate of the crosslinking reaction. Finally, the crosslinked PB sheet was obtained. Using differential scanning calorimetry (DSC), the glass transition temperature (*T*_g_) of the sample was determined to be 184 K.

### 2.2. Measurements and instrumentation

QEGS measurements using TDI (Baron *et al.*, 1997 ▸) were performed using a BL09XU instrument installed at the SPring-8 synchrotron radiation X-ray scattering facility in Nishiharima, Japan. The energy width of the incident radiation was set to ∼3.5 meV at the excitation energy of the first nuclear-excited state of ^57^Fe (14.4 keV) using a high-resolution monochromator comprising asymmetric Si (511) and Si (975) channel-cut crystals. The storage ring was operated in the several-bunch timing mode: 1/7-filling + several five-bunch mode with bunch intervals of 684.3 ns. The emitters of TDI were ^57^Fe-enriched (>96%) α-Fe foil. Full experimental details can be found in the literature (Saito *et al.*, 2017*b* ▸). Multi-element Si avalanche photodiode detectors were used to detect the scattered 14.4 keV γ-ray photons. In the QEGS measurements carried out under uniaxial deformation, the sample was stretched to the target value of strain γ = (*l* − *l*_0_)/*l*_0_, where *l*_0_ and *l* are the lengths of the samples before and after stretching, respectively, and stretching was maintained until the value of stress became constant. The samples were then exposed to X-ray irradiation for 2 h. In this sample system, the polymer chain moved to relax the applied stress, and the value of stress became constant after ∼1 h of stretching, thereby indicating that during the initial hour of stretching the state of the polymer chain changed continuously. Thus, the QEGS measurements commenced after reaching a constant stress value to ensure that the polymer dynamics were investigated under stable conditions. To study the temperature and strain dependences of the polymer dynamics under deformation, QEGS measurements were conducted at γ = 0.4 and 250 K, respectively. The detectors for the first peak of a structure factor of *q* = 14 nm^−1^ were set to be both parallel and perpendicular to the stretching direction to measure the intermolecular dynamics. In contrast, to measure the intramolecular dynamics, the detector for the second peak of a structure factor of *q* = 29 nm^−1^ was set only to perpendicular to the stretching direction owing to space limitations. DSC measurements were carried out using a Q200 calorimeter (TA Instruments) in the temperature range 123–423 K with a heating rate of 10 K min^−1^.

## 3. Results and discussion

**Figure 1:**
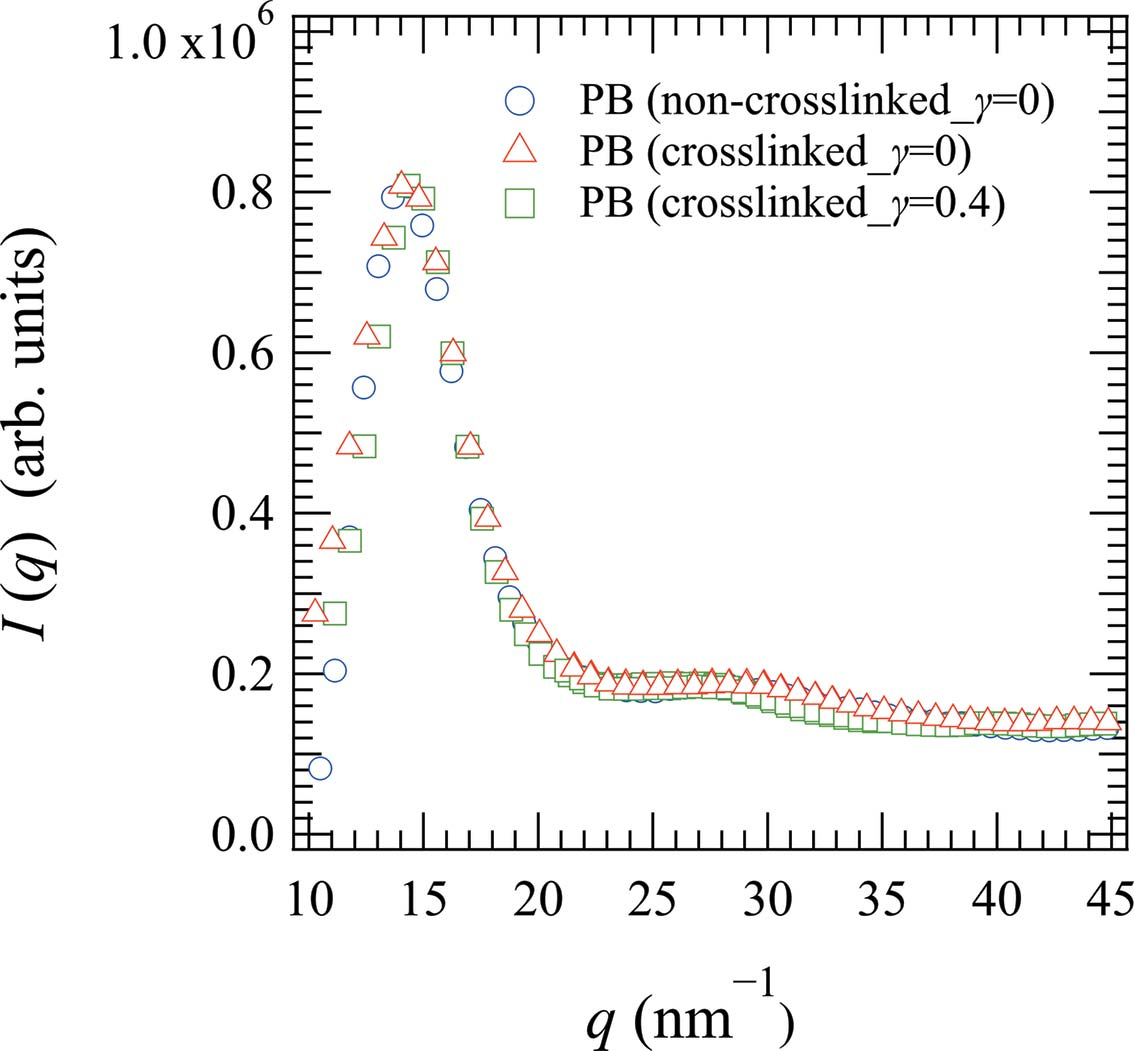
Scattering intensity profiles of the non-crosslinked PB at γ = 0 (blue circles) and the crosslinked PB at γ = 0 (red triangles) and 0.4 (green squares) at 303 K. The scattering intensity of the crosslinked PB at γ = 0.4 was measured in the direction perpendicular to elongation.

Fig. 1 ▸ shows the scattering intensity profiles of the non-crosslinked and crosslinked PB specimens in both the unstretched and stretched states at 303 K. The detector for the measurements of the scattering intensity profiles shown in Fig. 1 ▸ was set perpendicular to the stretching direction. The first peak of the structure factor originating from the intermolecular correlation of the polymer chain at *q* = 14 nm^−1^ and the second peak of the structure factor originating from the intramolecular correlation of the polymer chain at *q* = 29 nm^−1^ were also observed, and similar results were obtained by Kanaya *et al.* (2014 ▸) for non-crosslinked PB.

**Figure 2:**
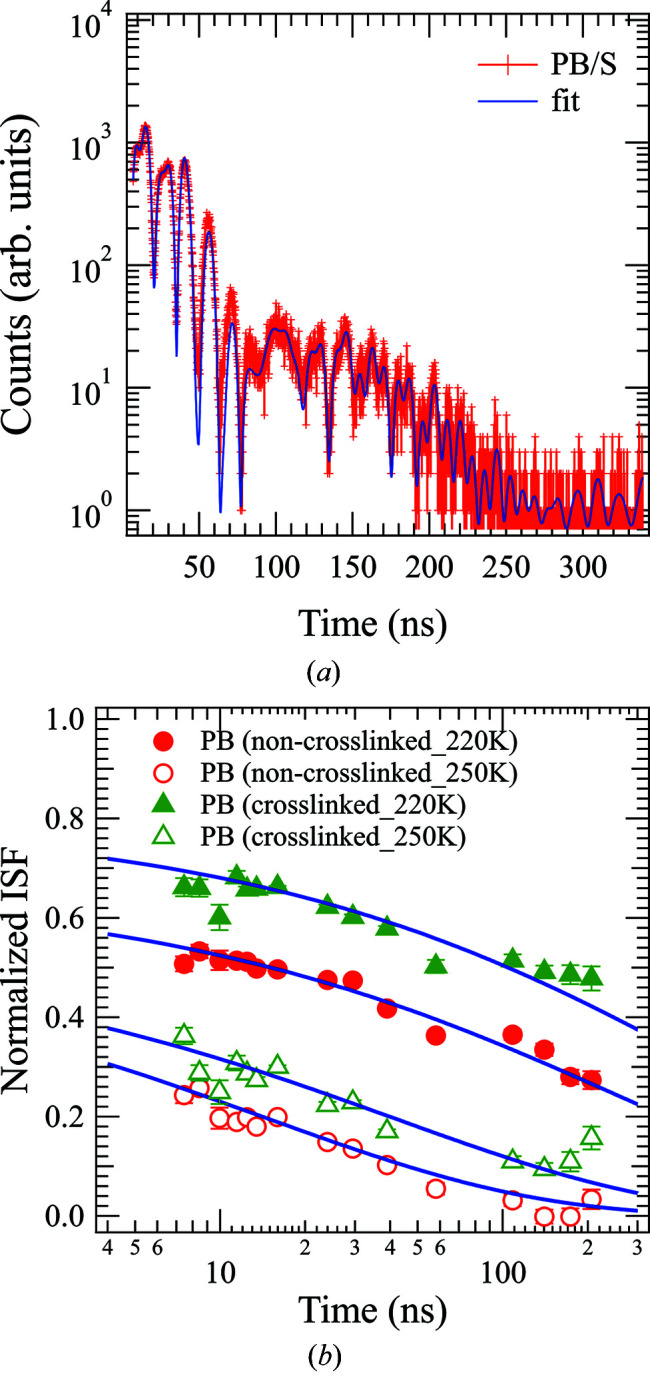
(*a*) Time spectrum of the crosslinked PB under uniaxial deformation at 250 K, and (*b*) the intermediate scattering functions of the non-crosslinked PB at 220 K (red filled circles) and 250 K (red open circles) and of the crosslinked PB at 220 K (green filled triangles) and 250 K (green open triangles) under uniaxial deformation in the direction parallel to elongation at *q* = 14 nm^−1^. The solid lines in (*a*) and (*b*) represent the fitting results.

Fig. 2 ▸ shows the time spectrum and intermediate scattering functions under uniaxial deformation, which were obtained by QEGS measurements. It should be noted here that the shape of the time spectrum is affected by the normalized intermediate scattering function of the sample, which contains dynamic information such as the relaxation time of the microscopic structures with a spatial scale corresponding to *q* (Saito *et al.*, 2017*b* ▸). The observed spectra were analyzed according to a previously described literature method (Saito *et al.*, 2017*b* ▸). More specifically, to analyze the non-crosslinked and crosslinked PB specimens, we assumed the Kohlrausch–Williams–Watts (KWW) function 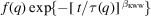 for the decay shape of the normalized intermediate scattering function, where *f*(*q*), β_KWW_ and τ are the relaxation amplitude, the stretching parameter and the relaxation time, respectively. In this context, we note that Richter *et al.* reported that the intermediate scattering function of non-crosslinked PB can be well described by the KWW function (Richter *et al.*, 1988 ▸, 2001 ▸). Furthermore, β_KWW_, which indicates the distribution of the relaxation time, was fixed at 0.45, based on the reports by Richter *et al.* Here, another report suggested that β_KWW_ = 0.41 (Arbe *et al.*, 1996 ▸), and the same tendency was observed in the following results based on fitting analysis with β_KWW_ = 0.41. As can be seen from Fig. 2 ▸, the time spectrum and intermediate scattering functions fit relatively well to the above-described method, thereby supporting the application of an appropriate model function. Despite the uncertainty of the fitting results because the QEGS data extended to approximately 300 ns, relaxation times exceeding 1000 ns as obtained by fitting analysis were considered to be reasonable within the error bar because the relaxation form was highly stretched and the QEGS data contained the edge of the longer relaxation component. Notably, neutron spin echo (NSE) studies based on similar analysis for polybutadiene (Richter *et al.*, 1992 ▸; Arbe *et al.*, 1996 ▸) also succeeded in determining relaxation times, which were much longer than the time window of observation. The obtained relaxation time by NSE is consistent with those obtained in other approaches, such as dielectric relaxation spectroscopy, and is useful in investigating the dynamics picture of the JG β-process.

**Figure 3:**
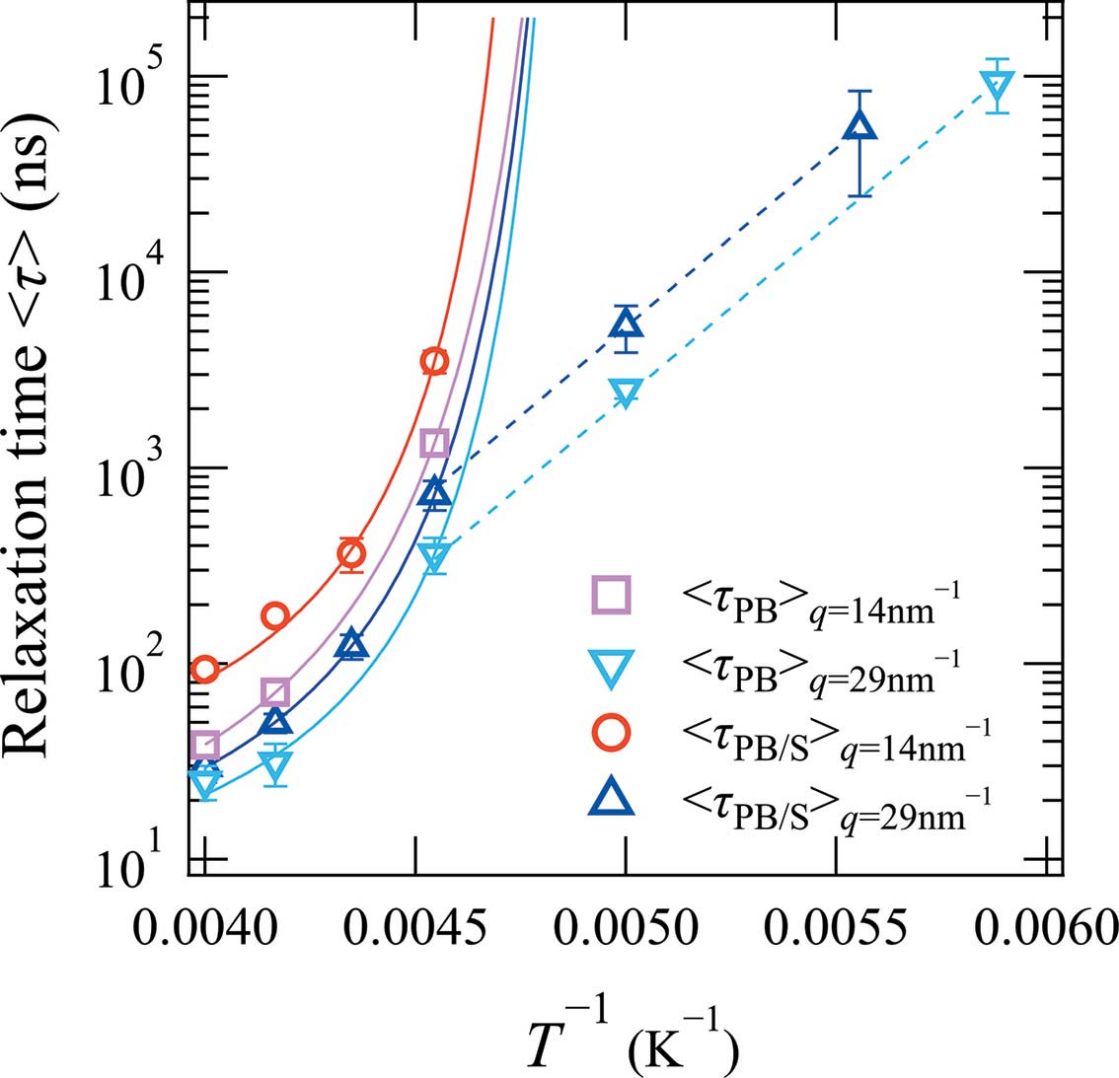
Temperature dependence of the mean relaxation times 〈τ〉 of the non-crosslinked and crosslinked PB samples in their unstretched states where *q* = 14 nm^−1^ (magenta squares, red circles) and 29 nm^−1^ (cyan down-triangles, blue up-triangles). The solid curves represent the results of fitting the data to the Vogel–Fulcher–Tammann equation, while the dashed lines represent the results of fitting the data to the Arrhenius equation, in which the activation energy of the JG β-process for the non-crosslinked PB (*E*_a_) is 35 kJ mol^−1^.

Fig. 3 ▸ shows the temperature dependence of the average relaxation time 〈τ〉 of the non-crosslinked and crosslinked PB specimens in their unstretched states where *q* = 14 or 29 nm^−1^. It should be noted that 〈τ〉 was calculated using 〈τ〉 = (τ/β)Γ(1/β), where Γ is a γ function (Kanaya *et al.*, 2014 ▸; Alvarez *et al.*, 1991 ▸). In Fig. 3 ▸, the solid curves are the results of fitting the data to the Vogel–Fulcher–Tammann (VFT) equation, namely 〈τ〉 = *A*_1_exp[*B*_1_/(*T* − *T*_0_)], where *T*_0_ is the temperature at which 〈τ〉 diverges (Vogel, 1921 ▸; Fulcher, 1925 ▸).

As indicated by the results presented in Fig. 3 ▸, the temperature dependences of 〈τ〉 for the non-crosslinked and crosslinked PB specimens in their unstretched states fit well with the VFT equation where *q* = 14 nm^−1^, which corresponds to the range of intermolecular correlation. This result indicates that cooperative segmental motion of the polymer chain, which is generally referred to as the α-process, took place in this spatial region. In addition, where *q* = 29 nm^−1^, which corresponds to the range of intramolecular correlation at *T* ^−1^ < 0.0045 K^−1^, the temperature dependences of 〈τ〉 also fit well with the VFT equation. Furthermore, this result shows that relaxation of the observed intramolecular motion at *q* = 29 nm^−1^ and *T* ^−1^ < 0.0045 K^−1^ is caused by the α-process, as in the case where *q* = 14 nm^−1^. On the other hand, where *q* = 29 nm^−1^ and *T* ^−1^ > 0.0045 K^−1^, the temperature dependences of 〈τ〉 did not fit well with the VFT equation, but instead exhibited a linear behavior, thereby corresponding to an Arrhenius-type temperature dependence. These observations therefore suggest that the JG β-relaxation process, which is perceived as the pre-motion of the α-process, is detected in this spatial and temperature range according to our previous report (Kanaya *et al.*, 2014 ▸). Thus, the mechanism of relaxation of the microscopic polymer motion depends on the temperature and *q* ranges (Saito *et al.*, 2012 ▸; Kanaya *et al.*, 2014 ▸). Moreover, the dashed line in Fig. 3 ▸ is the result of fitting the data to the Arrhenius equation {*i.e.* 〈τ〉 = 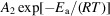 }, wherein the activation energy of the JG β-process for the non-crosslinked PB specimen (*i.e.* 35 kJ mol^−1^) was obtained by neutron spin echo measurements (Richter *et al.*, 1988 ▸, 1992 ▸).

According to the comparison of 〈τ〉 for the crosslinked and non-crosslinked PB specimens presented in Fig. 3 ▸, it was ascertained that three different relaxation times became longer following crosslinking: (i) the relaxation time of the intermolecular correlation induced by the α-process observed at *q* = 14 nm^−1^; (ii) the relaxation time of intramolecular correlation induced by the α-process observed at *q* = 29 nm^−1^, which corresponds to the intramolecular scale at *T* ^−1^ < 0.0045 K^−1^; and (iii) the relaxation time of intramolecular correlation induced by the JG β-process observed at *q* = 29 nm^−1^ at *T* ^−1^ > 0.0045 K^−1^. Hence, these results indicate that the microscopic molecular motion of the polymer chain is constrained by crosslinking, and the effect of crosslinking on the polymer dynamics can be successfully observed on the molecular scale using the QEGS method.

**Figure 4:**
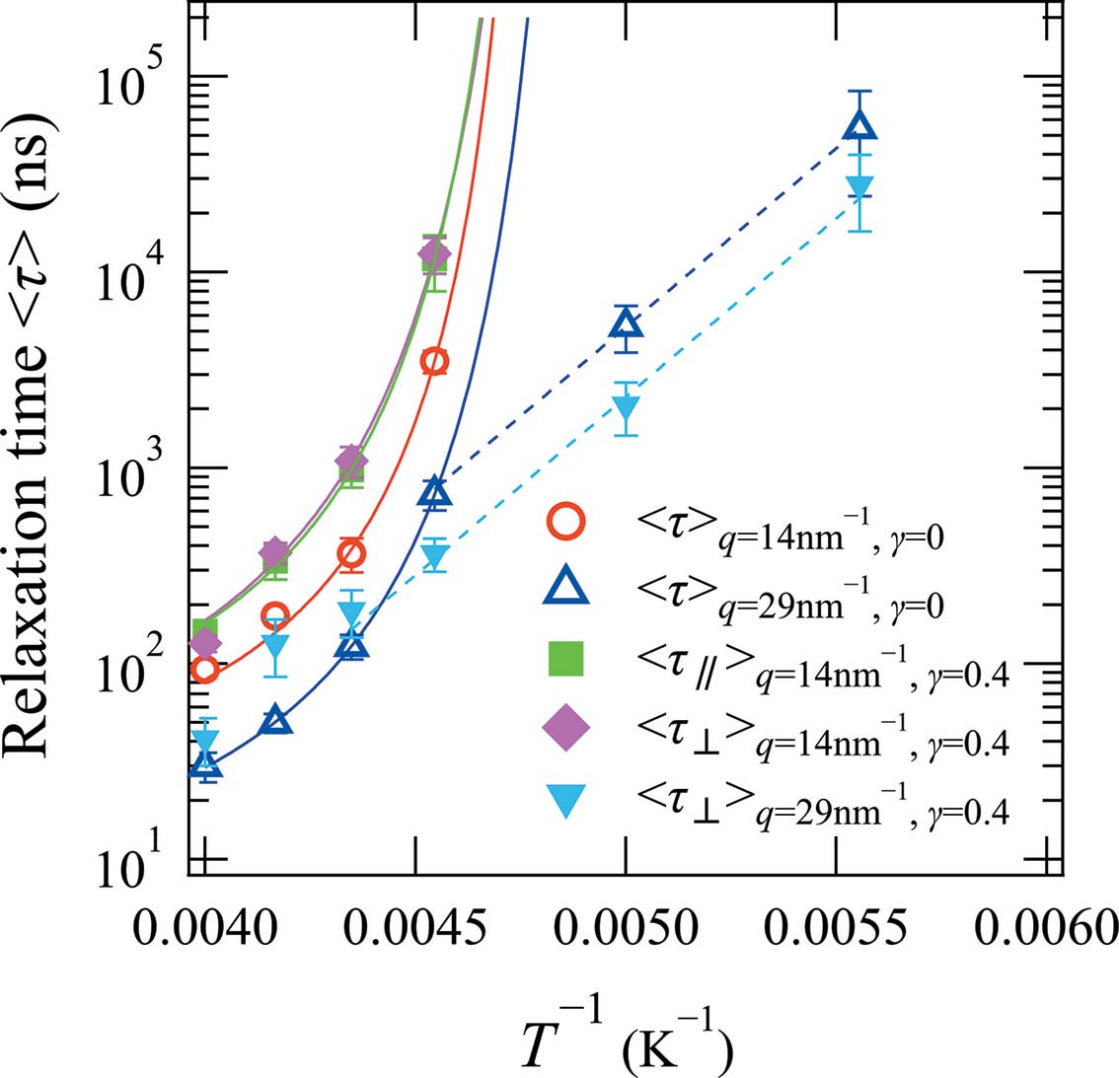
Temperature dependences of the mean relaxation times 〈τ〉 of the crosslinked PB in the unstretched state (γ = 0) at *q* = 14 nm^−1^ (red circles) and 29 nm^−1^ (blue open triangles), and under uniaxial deformation (γ = 0.4) at *q* = 14 nm^−1^ in the directions parallel (green filled squares) and perpendicular (magenta filled diamonds) to elongation. Also shown are the temperature dependences of the mean relaxation times 〈τ〉 of the crosslinked PB at 29 nm^−1^ in the direction perpendicular (cyan filled down-triangles) to elongation. The solid curves represent fitting of the data to the Vogel–Fulcher–Tammann equation. The dashed lines represent fitting of the data to the Arrhenius equation where the activation energy of the JG β-process for the non-crosslinked PB is 35 kJ mol^−1^.

Subsequently, we considered the polymer structure and dynamics under uniaxial deformation (γ = 0.4). As shown previously in Fig. 1 ▸, the first peak of the structure factor at *q* = 14 nm^−1^ and the second peak of the structure factor at *q* = 29 nm^−1^ are observed at both γ = 0 and 0.4, which indicates that elongation has little effect on the static structure. Thus, Fig. 4 ▸ shows the temperature dependence of the mean relaxation times 〈τ〉 of the crosslinked PB specimen in the unstretched state (γ = 0) and under uniaxial deformation (γ = 0.4). As shown, the temperature dependence of 〈τ〉 at *q* = 14 nm^−1^ and γ = 0.4 fit well with the VFT equation, as in the case where γ = 0. Furthermore, at *q* = 29 nm^−1^, *T* ^−1^ > 0.0045 K^−1^ and γ = 0.4, 〈τ〉 shows an Arrhenius-type temperature dependence, as also observed where γ = 0. These changes in 〈τ〉 that took place upon elongation can be summarized as follows: (i) the relaxation time of intermolecular correlation induced by the α-process observed at *q* = 14 nm^−1^ increases with elongation; (ii) the relaxation time of intramolecular correlation induced by the α-process observed at *q* = 29 nm^−1^ and *T* ^−1^ < 0.0045 K^−1^ increases with elongation; and (iii) the relaxation time of intramolecular correlation induced by the JG β-process observed at *q* = 29 nm^−1^ and *T* ^−1^ > 0.0045 K^−1^ decreases with elongation. These results therefore indicate that the microscopic relaxation time changes with the internal stress produced by elongation. Thus, the internal stress can be investigated using the relaxation time determined by the QEGS method.

It was not expected that the effects of elongation on the relaxation time would change based on the relaxation process, and in this study the effect of elongation on the JG β-process was observed on the microscopic molecular scale for the first time. It is therefore likely that the observed differences in the effects of elongation between the α-process and the JG β-process could provide additional clues to elucidate the origin of the JG β-process. In addition, there is little difference between the mean relaxation times in the directions parallel 〈τ_∥_〉 and perpendicular 〈τ_⊥_〉 to elongation; this result is also important for elucidating the mechanism of slowing the α-process relaxation time through elongation. Moreover, the factor of the KWW function *f*(*q*) does not change with the strain.

Subsequently, to elucidate the mechanism between stress concentration and fracture, the strain dependence of the relaxation time of polymer molecular motion was measured. From the viewpoint of the industrial application of crosslinked polymers, tyre rubber is generally used in relatively high temperature regions where the JG β-process does not take place. Therefore, the strain dependence of the relaxation time of the crosslinked PB was measured at 250 K, where polymer dynamics were observed on both the inter- and intramolecular scales. In other words, two strain dependences were successfully measured, namely the relaxation time at *q* = 14 nm^−1^ (corresponding to the intermolecular scale) and the relaxation time observed at *q* = 29 nm^−1^ (corresponding to the intramolecular scale).

**Figure 5:**
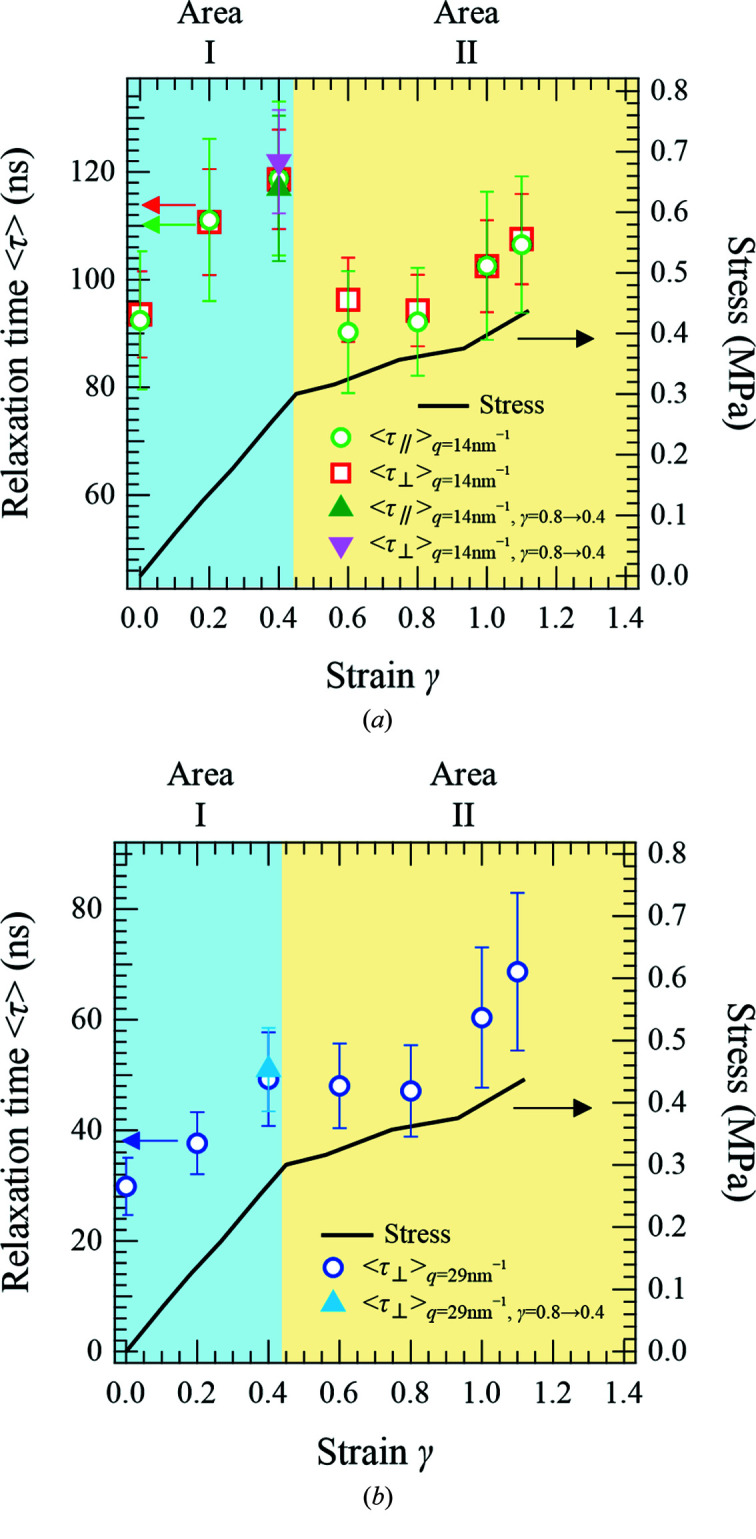
(*a*) Strain dependences of the mean relaxation times 〈τ〉 at *q* = 14 nm^−1^ in the directions parallel (green open circles, green filled up-triangles) and perpendicular (red open squares, magenta filled down-triangles) to elongation of the crosslinked PB. (*b*) Strain dependences of the mean relaxation times 〈τ〉 at *q* = 29 nm^−1^ in the direction perpendicular to elongation (blue open circles, cyan filled up-triangle) of the crosslinked PB. The cyan filled up-triangles, the magenta filled down-triangles and the green filled up-triangles represent the values of 〈τ〉 when the crosslinked PB was stretched to γ = 0.8 and then deformed to γ = 0.4. The black solid lines represent the stress–strain curves.

Fig. 5 ▸ shows the strain dependences of the mean relaxation times 〈τ〉 and the stresses of the crosslinked PB specimen at *q* = 14 and 29 nm^−1^. Thus, the results of the dynamic measurements corresponding to these conditions are discussed in terms of Area I (*i.e.* the elastic response regime, 0 < γ < 0.45) and Area II (*i.e.* the non-elastic response regime, γ > 0.45). As shown in Fig. 5 ▸(*a*), no clear difference was observed between the mean relaxation times in the directions parallel 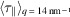 and perpendicular 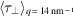 to elongation for either area in the case where *q* = 14 nm^−1^. However, where *q* = 14 and 29 nm^−1^, 〈τ〉 increased with increasing stress in Area I, then decreased prior to increasing once again in Area II. Here, the decrease in 〈τ〉 is smaller at *q* = 29 nm^−1^ than at *q* = 14 nm^−1^ in the γ range of 0.4–0.6. However, based on the error range for the data, it was considered that the behaviors of 〈τ〉 at *q* = 29 and 14 nm^−1^ were comparable. Although the error bars of 〈τ〉 were relatively large as seen in Fig. 5 ▸, the reproducibility of these results was already confirmed. It should also be noted here that the decrease in 〈τ〉 in Area II despite the increase in stress is noteworthy. Furthermore, in Area I, the observed increase in 〈τ〉 with a greater strain indicates that the configurational entropy decreases due to the constraint of polymer chain motion under stretching conditions (Choi *et al.*, 2012 ▸). The mechanism responsible for these observations was considered to be comparable with that described above to account for the longer relaxation time of the crosslinked PB compared with the non-crosslinked PB. Moreover, in Area II, it was assumed that 〈τ〉 decreases due to the partial release of constraint in the polymer chain motion, which in turn is caused by unraveling of the polymer chain entanglement (Yashiro *et al.*, 2003 ▸; Mahajan *et al.*, 2010 ▸) or the occurrence of nano-voids (Payal *et al.*, 2019 ▸), as predicted by molecular dynamics simulations. Another interpretation is that the degree of polymer fracture occurring under elongation conditions accelerates the molecular dynamics. Thus, to reveal whether the polymer chains were broken during elongation, the polymer dynamics were measured again in Area I (at γ = 0.4) after previous stretching to the Area II region (γ = 0.8). As shown in Fig. 5 ▸, the effect of polymer chain fracture during elongation is undetectable. Finally, in the final phase of Area II (γ > 0.95), the polymer chain was further enlarged between the crosslinks, resulting in significantly constrained polymer chain motion, thereby accounting for the further increase in 〈τ〉 with increasing strain in this region. Although the interpretation of the results of strain dependence of 〈τ〉 remains unclear, these unique results support the solution to the unresolved problem of microscopic explanation in stress-proceeding phenomena.

## 4. Conclusion

We developed a method for the measurement of polymer dynamics under uniaxial deformation, which was based on the use of quasi-elastic γ-ray scattering with time-domain interferometry to experimentally determine the stress-producing mechanism of crosslinked polybutadiene on a molecular scale. Initially, we evaluated the effect of elongation on the temperature dependence of the mean relaxation time 〈τ〉 of the microscopic polymer motion. It was found that the relaxation time observed at *q* = 14 nm^−1^ (corresponding to the intermolecular scale) and the relaxation time observed at *q* = 29 nm^−1^ (corresponding to the intramolecular scale at *T* ^−1^ < 0.0045 K^−1^) increased with elongation, while the relaxation time observed at *q* = 29 nm^−1^ and *T* ^−1^ > 0.0045 K^−1^ decreased with elongation. In addition, we evaluated the effect of elongation on the strain dependence of the relaxation time of the polymer molecular motion to elucidate the mechanism between stress concentration and fracture at 250 K. As a result, two key areas were identified where the slope indicating the strain–stress relationship increased in gradient, *i.e.* Area I (0 < γ < 0.45) and Area II (γ > 0.45). In the cases where *q* = 14 and 29 nm^−1^, 〈τ〉 increased sharply with an increased stress in Area  I, but decreased in the early part of Area II, prior to increasing sharply once again. These results therefore indicate that our method allowed evaluation of the local molecular dynamics of polymer chains under uniaxial deformation on both the inter- and intramolecular scales separately for the first time. Since the relaxation time obtained herein can also be calculated using molecular dynamics simulations, it is possible to understand the origin of stress production by comparing the results of the molecular dynamics simulations with the results obtained experimentally. We therefore believe that this study represents a major breakthrough in allowing the experimental elucidation of stress-producing phenomena on the molecular scale.
